# The mediating role of prenatal depression in adult attachment and maternal-fetal attachment in primigravida in the third trimester

**DOI:** 10.1186/s12884-021-03779-5

**Published:** 2021-04-16

**Authors:** Ling Zhang, Lei Wang, Qiuyu Yuan, Cui Huang, Shu Cui, Kai Zhang, Xiaoqin Zhou

**Affiliations:** 1 School of Mental Health and Psychological Sciences, Anhui Medical University, Hefei, 238000 China; 2 Chaohu Hospital, Anhui Medical University, Hefei, 238000 China

**Keywords:** Prenatal depression, Primigravida, Adult attachment dimension, Maternal-fetal attachment

## Abstract

**Background:**

Prenatal depression and adult attachment are factors that affect the establishment of an intimate relationship between a mother and fetus. The study explored differences in prenatal depression and maternal-fetal attachment (MFA) scores between different types of adult attachment and the effects of maternal depression scores and attachment dimensions on maternal intimacy with the fetus.

**Methods:**

The Edinburgh Postnatal Depression Scale (EPDS), Experience of Close Relationship (ECR) scale, Maternal Antenatal Attachment Scale (MAAS) and a general data scale were used to investigate 260 primigravida. An exploratory analysis was performed to analyze the effects of the depression score and adult attachment on MFA.

**Results:**

The results showed that pregnant women with insecure attachment exhibited an increased prevalence of prenatal depression, lower total MFA scores, and lower MFA quality compared with those women with secure adult attachment. The explorative analysis showed that the depression scores mediated the relationship between adult attachment avoidance and MFA quality.

**Conclusions:**

Primigravida who had insecure adult attachment exhibited an increased prevalence of prenatal depression and lower MFA. Maternal depression and adult attachment may affect the emotional bond between a mother and fetus. This finding should be seriously considered, and timely intervention needs to take personality traits into consideration.

**Supplementary Information:**

The online version contains supplementary material available at 10.1186/s12884-021-03779-5.

## Background

Pregnancy is a significant and arduous process that can be extremely challenging for a woman physiologically and psychologically. The prevalence of perinatal depression in the Chinese population ranges between 15 and 20% [1], and the prevalence among women in low- and middle-income countries/regions is significantly higher [2]. A systematic review involving 48,904 persons from 20 low-income middle-income countries reported that the prevalence of depression was 25.3% prenatally [3]. Prenatal depression not only affects the mood of pregnant women, such as increasing the risk of suicide and the prevalence of postpartum depression [4, 5], but also affects the development of the fetus [6, 7], and even increases the risk of mental health issues in the child [8]. Moreover, prenatal depression can significantly predict the degree of attachment of pregnant women to the fetus [9, 10] and even affect the intimate bond with the child after pregnancy [11].

Maternal-fetal attachment (MFA) is the mother’s emotional connection to the fetus and is an important indication of whether the pregnant mother can adapt to changes in pregnancy and assume maternal responsibility [12]. MFA increases with gestational age and stabilizes in the third trimester [13]. In addition, MFA can predict the postpartum mental status of mothers, such as the presence of anxiety and depression, and affect the postpartum mother-infant attachment quality [14]. Pregnant women with weak MFA are less likely to engage in health promotion activities and are more likely to have an unhealthy newborn than those with strong MFA [8].

Moreover, a significant positive correlation is noted between MFA and neonatal outcome during pregnancy [15]. In addition, the level of MFA can predict children’s development of early behavioral and emotional ability [16]. Furthermore, a couple’s relationship is among the most important predictors of MFA in addition to the mental health of pregnant women [17]. The mental health of her partner affects how a pregnant woman feels about the fetus. For example, when a partner’s childcare pressure is high, the intimate relationship between a woman and her child during the perinatal period will decrease [18]. In addition to prenatal depression, many factors are predictors of MFA, such as the gestational age, social support and prenatal testing [19]. In addition, education level, obstetric problems during pregnancy, duration of pregnancy, socioeconomic status, fertility attitude, and fetal awareness are factors that affect MFA [20, 21]. The personality characteristics of pregnant women, such as adult attachment, can also affect MFA.

Attachment is defined as an emotional connection between an individual and their primary caregiver during the earliest stage of life [22]. Attachment behavior forms an internal working model during individual development. Adult attachment is the continuation and reappearance of early attachment experience. When people encounter difficulties or significant changes in life, such as pregnancy [23], the internal working model is activated and manifested in different ways, eliciting emotional and behavioral responses. Adult attachment plays an acknowledged role in maternal mental health [24]. Catherine’s quality-stress model showed that relationship templates dominated by fear or lack of security, such as insecure attachment, may become a personality trait rendering women more vulnerable to perinatal depression than those without this trait [25]. The level of prenatal attachment affects maternal-infant attachment after delivery [26]. In a recent study by Huang and colleagues, women with greater adult attachment anxiety and avoidance exhibited more symptoms of depression [27].

Studies have shown that primiparas have a higher incidence of prenatal depression and anxiety scores after pregnancy than multiparas [28]. Due to the lack of fertility experience and low sense of self-efficacy in parenting [29], there are certain obstacles to their development of the mother-fetal relationship, indicating that special attention should be paid to primiparas.

Taken together, prenatal depression, MFA and adult attachment all are related. A previous study indicated that depression partially mediated the connection between insecure attachment and mother-infant attachment after birth [30]. Additionally, the regulatory role of postpartum depression symptoms in the relationship between different attachment types and postpartum bonding has been confirmed [31]. However, the role of maternal depression and two dimensions of attachment (avoidance and anxiety) in MFA during pregnancy are currently unknown. This article aimed to explore the difference between prenatal depression and MFA in pregnant women with different attachment styles and the role of maternal depression and attachment on MFA in primiparas. More specifically, we predicted that maternal depression mediates the relationship between the two dimensions of attachment and MFA.

## Methods

### Procedure and participants

The data were collected at the antenatal clinic of Chaohu Hospital of Anhui Medical University. The hospital is a comprehensive tertiary hospital that provides medical services to approximately one million people. The average number of deliveries in the hospital is approximately 2000 per year. The inclusion criteria were as follows: 1) primigravida; 2) pregnant women aged 18–45 years; 3) women with a gestational age of 28–40 weeks; and 4) women with a singleton gestation. The exclusion criteria were as follows: 1) a previous history of mental illness; 2) a high-risk pregnancy (gestational diabetes, hypertension and preeclampsia); and 3) a history of miscarriage.

Before the start of the study, three graduate students and two nurses received unified standardized training regarding the entire research process, the precautions necessary while completing the self-made general information questionnaire, and the unified instructions of each scale. The training time was approximately two weeks. Data collection was based on convenience sampling along with probability sampling. We randomly selected the time from September to December 2019 to collect samples. According to the pregnant women’s consultation number, those with an odd number of consultation numbers were randomly selected to fill out all the scales. Finally, according to the inclusion and exclusion criteria, a total of 260 samples that met the criteria were included. Before completing the questionnaire, all participants were instructed by standardized and uniformly trained staff. Approximately ten minutes were required to complete all questionnaires. The ethics committee of Chaohu Hospital of Anhui Medical University approved the study protocol. The procedures used in this study adhered to the principles of the Declaration of Helsinki. All women signed informed consent forms before participating in this study.

### Measures

#### Demographic characteristics

We used a self-designed questionnaire to collect the demographic characteristic data, including age, gestational age, education level, planned pregnancy, prenatal education, working status, exercise, and marital satisfaction of the pregnant women enrolled in our study (the specific content of the self-designed questionnaire is shown in the supplementary material file).

#### Prenatal depression

The Edinburgh Postpartum Depression Scale (EPDS) [32] was chosen to assess the participants’ severity of depression. The EPDS can be used to screen for postpartum depression as well as depression during pregnancy. The EPDS contains a total of 10 items. The total score ranges from 0 to 30, and the higher the score is, the more serious the degree of depression. Cronbach’s alpha was 0.78 and test-retest reliability was 0.90 [33]. We used a cut-off of ≥12 in this study. We used the Chinese version of the EPDS, which has been verified and frequently used in previous studies [34–36].

#### Adult attachment

Adult attachment in all pregnant women was assessed with the Experience of Close Relationship (ECR) scale [37], which has demonstrated high measurement accuracy [38]. The reliability and validity of this scale have been tested in China [39], and it has been used in the Chinese population [40]. The scale consists of 36 items, each ranging from 1 “strongly disagree” to 7 “strongly agree”. The scale has the following two dimensions: anxiety and avoidance. The avoidance subscale includes 18 items, indicating the avoidance of intimacy and interdependence. The anxiety subscale also includes 18 items and indicates concerns about exclusion and abandonment. According to the score of the two dimensions, adult attachment can be divided into secure and insecure attachment, and there are three types of insecure styles (attentive, indifferent and phobic).

#### MFA

The Maternal Antenatal Attachment Scale (MAAS) [41] was used to assess the MFA of the participants. The reliability and validity of this scale have been tested in Chinese pregnant women [42]. The MAAS is a self-reported scale that includes 19 questions with a 5-point scoring system, and the total MFA score ranges from 5 to 95, with higher score signifying higher MFA [43]. The scale includes the following two sub-dimensions: “MFA quality” (items 3, 6, 9, 10, 11, 12, 13, 15, 16, and 19) and “MFA intensity” (items 1, 2, 4, 5, 8, 14, 17, and 18). Item 7 is only included in the total score and does not affect any of these two dimensions. The MFA quality indicates the emotional experience with regard to the fetus, and the MFA intensity indicates the time and energy devoted to the fetus by the pregnant women.

#### Data analysis

We used the Statistical Package for Social Sciences (IBM SPSS 22.0) for all analyses conducted in this study. The continuous variables were tested by t-test or Mann-Whitney U test according to whether they exhibited a normal distribution, and the chi-square test was used to classify the variables. Before the mediation analysis, Spearman’s correlation was calculated to determine the correlations between attachment anxiety/avoidance, the maternal depression score, and MFA. Finally, we found pairwise correlations between anxiety/avoidance, the depression score, and the MFA quality. Subsequently, model 4 of Hayes’s PROCESS macro and Bootstrap were used to analyze the mediating effect [44]. The model estimates the direct effect of anxiety/avoidance on maternal depression and maternal depression on MFA, the indirect effect of attachment anxiety/avoidance on MFA mediated by maternal depression as well as the direct effect of on MFA. A *p*-value of 0.05 was considered to be statistically significant.

## Results

### Demographic characteristics of the participants

Two hundred and sixty pregnant women were enrolled in our study. The mean age of the pregnant women was 26.52 (SD = 3.18) years, and the mean gestational age was 35.57 (SD = 2.57) weeks. Among all participants, 54.23% of the pregnant women had secure adult attachment. Most women had a high school or junior college diploma (51.93%), and those with an education level of junior high school or lower and bachelor’s degree or higher accounted for 16.15 and 31.92% of the total, respectively. Moreover, 71.15% of the participants had a planned pregnancy, and 64.23% of the participants received prenatal education during pregnancy. In addition, 59.62% of the pregnant women were employed. When asked whether they exercised during the pregnancy period, 71.15% of the pregnant women said yes. Additionally, 84.23% of the participants reported that they were satisfied with their marriage. Our results also showed that in total, 18.85% of the pregnant women had a score of 12 or greater, indicating prenatal depression. The proportion of women with low MFA (Total MAAS ≤75) was 55%. The mean total MFA score was 74.12 (SD = 7.32), and the mean MFA intensity and MFA quality were 28.21 (SD = 4.62) and 42.14 (SD = 4.57), respectively.

### Differences in the demographic characteristics, prenatal depression and MFA between the secure and insecure groups

As shown in Table 1, compared with the secure group, the prevalence of depression was increased in the insecure group (25.21% vs 13.48%, *p* = 0.016). The EPDS score was also higher in the insecure group (8.64 ± 4.47 vs 6.99 ± 4.24, *p* = 0.003). In addition, the proportion of women with low MFA was higher in the insecure group (62.18% vs 48.94%, *p* = 0.032). Furthermore, the results suggested that the total MFA score and MFA quality in the insecure group were both lower than those in the secure group (72.87 ± 7.14 vs 75.17 ± 7.33, *p* < 0.001, 41.34 ± 4.48 vs 42.82 ± 4.54, *p* = 0.003). Moreover, no differences in the demographic characteristics and MFA intensity were noted between the two groups (all *p* > 0.05).

**Table 1:** Demographic characteristics, prenatal depression and MFA between the secure and insecure groups

	All participants (*n* = 260)		Secure (*n* = 141;54.23%)	Insecure (*n* = 119;45.77%)	t/Z/X^2^	P
Age (years)	26.52 ± 3.18		26.66 ± 3.03	26.34 ± 3.37	−1.020	0.308
Gestational weeks	35.57 ± 2.57		35.37 ± 2.73	35.81 ± 2.35	−0.1056	0.291
Education						
High school or lower		42 (16.15%)	19 (13.48%)	23 (19.33%)	4.893	0.087
High school or junior college		135 (51.93%)	82 (58.16%)	53 (44.54%)		
Bachelor degree or higher		83 (31.92%)	40 (28.37%)	43 (36.13%)		
Planned pregnancy	Yes	185 (71.15%)	98 (69.50%)	87 (73.11%)	0.409	0.523
Prenatal education	Yes	167 (64.23%)	95 (67.38%)	72 (59.66%)	1.326	0.249
Employed	Yes	155 (59.62%)	57 (50.00%)	98 (82.35%)	< 0.001	0.988
Exercise	Yes	185 (71.15%)	98 (69.50%)	87 (73.11%)	0.409	0.523
Marital satisfaction	Yes	219 (84.23%)	123 (87.23%)	96 (80.67%)	2.092	0.148
Prenatal depression	Yes	49 (18.85%)	19 (13.48%)	30 (25.21%)	5.811	**0.016**
EPDS score	7.75 ± 4.14		6.99 ± 4.24	8.64 ± 4.47	−2.922	**0.003**
Low MFA	143 (55%)		69 (48.94%)	74 (62.18%)	4.577	**0.032**
Total score of MFA	74.12 ± 7.32		75.17 ± 7.33	72.87 ± 7.14	2.548	**< 0.001**
MFA intensity	28.21 ± 4.62		28.49 ± 4.39	27.87 ± 4.87	−1.533	0.125
MFA quality	42.14 ± 4.57		42.82 ± 4.54	41.34 ± 4.48	−2.992	**0.003**

### Explorative analysis

**Fig. 1:**
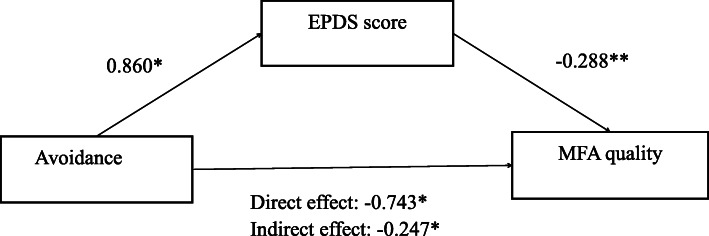
Results of mediating analysis showing associations between avoidance (independent variable), EPDS score (mediator), and MFA quality (outcome variable). Legend: EPDS, Edinburgh Postnatal Depression Scale; *p < 0.05, **p < 0.01

To determine the relationship among the variables of the EPDS score, two dimensions of adult attachment (avoidance and anxiety) and MFA, Spearman’s correlation analyses were performed (Table 2). As shown in Table 2, both avoidance and anxiety were correlated with the EPDS score and the MFA quality (all *p* < 0.05), and the EPDS score was correlated with the MFA quality. Based on these results, an SPSS process script was used to analyze the mediating role of the EPDS score in the relationship between avoidance (anxiety) and the MFA quality. However, there was only a significant indirect effect of avoidance on the MFA quality through depression (b = − 0.2474, SE = 0.1175, 95% CI = [− 0.5438–-0.0691]). Figure 1 shows the coefficients of the relationships among the independent, mediating, and outcome variables.

**Table 2:** Correlation analysis results of each variable

Variable	EPDS score	Avoidance	Anxiety	Total score of MFA	MFA intensity	MFA quality
EPDS score	1.000					
Avoidance	0.171^**^	1.000				
Anxiety	0.330^**^	0.245^**^	1.000			
Total score of MFA	−0.044	−0.192^**^	−0.023	1.000		
MFA intensity	0.101	−0.143	0.059	0.835^**^	1.000	
MFA quality	0.235^**^	−0.194^**^	−0.144*	0.794**	0.410**	1.000

Legend: *p < 0.05, **p < 0.01

## Discussion

This is the first study on the associations between of attachment and EPDS score on MFA in primigravida to the best of our knowledge. The transition to motherhood implies great changes in the role and life of pregnant women; thus, helping pregnant women through pregnancy can promote a good pregnancy outcome. These results are helpful for understanding the influence of adult attachment and depression on MFA.

In our study, in total, 18.85% of the participants suffered from prenatal depression, which was lower than the incidence reported in another study involving Chinese primipara in late pregnancy [45]. A possible explanation is a difference in the prenatal depression screening scale used, and another possible explanation might be the difference in the demographic data. A study conducted in South African showed that the prevalence of prenatal depression was 21% [46]. The prevalence of prenatal depression was 27.5% among Turkish Women [47], 10.0% during late pregnancy in the Netherlands [48], 14.8% in Spain [49], and 6.1% in the US [50]. These results also indicate that there are differences in the prevalence of prenatal depression among pregnant women from different cultures.

We found that women with secure adult attachment had a lower prevalence of prenatal depression. A pregnant woman’s attachment patterns continue to affect her experience during pregnancy, increasing the risk of mood disorders during pregnancy [25]. Individuals with secure adult attachment seek social support to regulate negative emotions when needed [51]. Moreover, pregnant women with secure adult attachment reported a lower proportion of low MFA, a high total MFA score and a high MFA quality. A study involving 165 pregnant women in Poland also found that maternal attachment affects women’s image as a mother and their connection with their fetus [52], and people with secure adult attachment are more likely to assume the role of parents [23]. Therefore, prenatal intervention can be performed in terms of the personality structure of the pregnant women, such as adult attachment. A previous study also noted that it is necessary to screen for attachment styles and provide tailored care during pregnancy [53]. But there was no difference in MFA intensity between the secure attachment and the insecure attachment groups. Studies have also pointed out that the quality of maternal-fetal attachment is affected by attachment-related avoidance, while the intensity of maternal-fetal attachment is not affected by adult attachment, which also showed that the intensity and quality dimensions need to be addressed individually [17].

Further analysis revealed that maternal attachment avoidance and anxiety were negatively related to the MFA quality, which is consistent with previous research results [54]. Self-reported romantic attachment predicts high avoidance scores using the parental role scale in pregnant women, and these women may face difficulties in developing their maternal identity [52]. Those who attached great importance to adult attachment avoidance felt uncomfortable with intimacy and invalidated the attachment system [55]. Women with high avoidance may regard pregnancy and the fetus as sources of pain and, thus, adopt a strategy of alienating the fetus and avoiding emotional involvement [56]. As a result, the emotional experience with regard to the fetus may be relatively low. In addition, people with high adult attachment anxiety are afraid of rejection. Thus, these individuals may think that they do not have the ability to cultivate intimate relationships and pay more attention to their own distress and attachment needs [57, 58]. There was also a negative correlation between depression during pregnancy and the MFA quality. Previous research also revealed that pregnant women with depression were less sensitive to the fetus [59] and that depression during pregnancy was also a risk factor for poor MFA [12].

The exploratory mediation analysis revealed that low maternal adult attachment avoidance directly and indirectly reduced the MFA quality score through the prenatal depression score. The results suggested that an avoidance attitude toward the attachment subjects rendered the women susceptible to the interference of depression symptoms, reducing emotional investment in the fetus. A possible mechanism is that the internal working model of attachment has an important impact on an individual’s cognition, emotion, and behavior with regard to interpersonal communication, and the avoidance attachment model formed in childhood leads to a sense of mistrust of others in pregnant women. This internal working model is triggered by stressful events, such as pregnancy, and leads to depression during pregnancy, further reducing the emotional investment and energy devoted to the fetus, that is, poor MFA. Condon indicated that parental psychological variables could affect the MFA quality. Moreover, attachment avoidance-related deactivation strategies are associated with depression maintenance [14]. In fact, when perceiving positive emotional information, people with high attachment avoidance are unable to experience positive emotions; to avoid the activation of the attachment system, they tend to deactivate their emotional channels and hide their feelings [60]. People with a high level of avoidance are less willing to be parents and feel more pressure to raise children [61]. It seems that adult attachment avoidance and the prenatal depression score both affect the mother’s intimate connection with the unborn fetus. Another study involving high-risk pregnant women also showed that higher types of avoidant attachment were associated with a lower quality of maternal-fetal bonding. However, inconsistently, in our findings, when depressive symptoms were entered into the regression model, the effect of avoiding attachment was no longer significant [62]. The possible reasons are related to the different samples selected. Therefore, the relationship among these three variables needs to be further studied during different pregnancy periods and in different subgroups of pregnant women in the future. However, no mediating role of the prenatal depression score was observed in the relationship between adult attachment anxiety and the MFA quality, and a previous study demonstrated that caregivers with a higher degree of avoidance are less responsive than those with a lower degree of avoidance [63]. The MFA quality is related to the mental health of mothers, whereas the MFA intensity is not associated with mental health [64]. Existing research has also suggested that future research should focus on the dimensions of MFA.

Our results revealed the relationship between adult attachment avoidance and the prenatal depression score and their effects on the degree of emotional communication and interaction between the mother and the fetus, further providing a basis for understanding the development of maternal-fetal relationships. Therefore, to improve the MFA of pregnant women, we can start from the personality characteristics of pregnant women, such as screening for insecure attachment or high avoidance attachment during antenatal examinations and promoting a positive model for pregnant women to target others and themselves. In addition, for pregnant women with poor mental health, such as high depression scores, family members can increase their social support and health care institutions should popularize knowledge regarding pregnancy to reduce the negative effects of pregnancy. Studies have also noted that by determining the mother’s attachment style and MFA status during pregnancy, the early detection of these problems provides an opportunity for professionals to offer specific support and individualized interventions to improve these interactions [65]. In prenatal screening, the timely detection of low MFA and formulating corresponding interventions can improve the quality of life during pregnancy and childhood [20]. Pregnancy adaptation education can improve not only maternal-fetal attachment but also pregnant women’s ability to adapt to pregnancy [66]. Some studies also believe that detecting mental well-being, such as depression symptoms and formulating corresponding interventions can help improve MFA [67]. In addition, it is also important to increase support for pregnant women and raise the attention of society and families to these issues [68]. Culture as a macro factor may affect the level of maternal-fetal attachment. The researchers also noted the need to raise awareness of cultural influences when evaluating MFA [69]. In addition, a previous study showed that culture has an influence on the depression and attachment dimension [70]. In the future, we need to explore maternal-fetal attachment from the Chinese cultural background perspective, such as the cultural perspective of collectivism with dense social networks and the former one-child policy.

There are some limitations in our study. First, this study was restricted by its cross-sectional design, and we did not track postpartum depression and mother-infant attachment, which should be evaluated in the next steps of our work. Second, given that all data were evaluated at the measurement point, directional and causal conclusions cannot be drawn directly given this design. Moreover, the data collection was based on convenience sampling; thus, our participants may not represent the general pregnant population. In addition, all participants were in the third trimester of pregnancy; thus, the results obtained in this particular group may not easily generalize to pregnant women of other gestational weeks. Pregnant women of other gestational weeks need to be included in future studies.

Despite these limitations in our research, our findings provide new insight into understanding the mother-fetal relationship before childbirth and might be effective in providing guidance for prenatal psychological education and individualized interventions.

## Supplementary Information

**Additional file 1.** General Demographic Information Questionnaire.

## Data Availability

All data supporting our findings are presented in the manuscript; the datasets used and/or analyzed during the current study are available from the corresponding author upon reasonable request.

## Abbreviations

EPDS: Edinburgh Postpartum Depression Scale
ECR: Experience of Close Relationship
MAAS: Maternal Antenatal Attachment Scale
MFA: Maternal-fetal attachment
CI: Confidence interval
