# Supplementary material for: The mediating role of prenatal depression in adult attachment and maternal-fetal attachment in primigravida in the third trimester
Source: BMC Pregnancy Childbirth. 2021 Apr 16;21:307. doi: 10.1186/s12884-021-03779-5 (PMC8052769; doi:10.1186/s12884-021-03779-5)
Supplement: Supplementary file 1 — Additional file 1. General Demographic Information Questionnaire. [file 12884_2021_3779_MOESM1_ESM.doc]

General Demographic Information Questionnaire

Q1 How old are you now？

Q2What is your gestational age?

Q3.What is your education level？

■High school or lower

■High school or junior college

■Bachelor degree or higher

Q4.Is this pregnancy planned?

■Yes

■No

Q5. Have you received prenatal education?

■Yes

■No

Q6. Are you employed during pregnancy?

■Yes

■No

Q7. Do you exercise regularly during pregnancy?

■Yes

■No

Q8. Are you satisfied with your marriage?

■Yes

■No
